# Supplementary material for: Neither Trimethylamine-N-Oxide nor Trimethyllysine Is Associated with Atherosclerosis: A Cross-Sectional Study in Older Japanese Adults
Source: Nutrients. 2023 Feb 2;15(3):759. doi: 10.3390/nu15030759 (PMC9921512; doi:10.3390/nu15030759)
Supplement: Supplementary file 1 [file nutrients-15-00759-s001.zip › nutrients-2191597-Supplementary Figure.pdf]

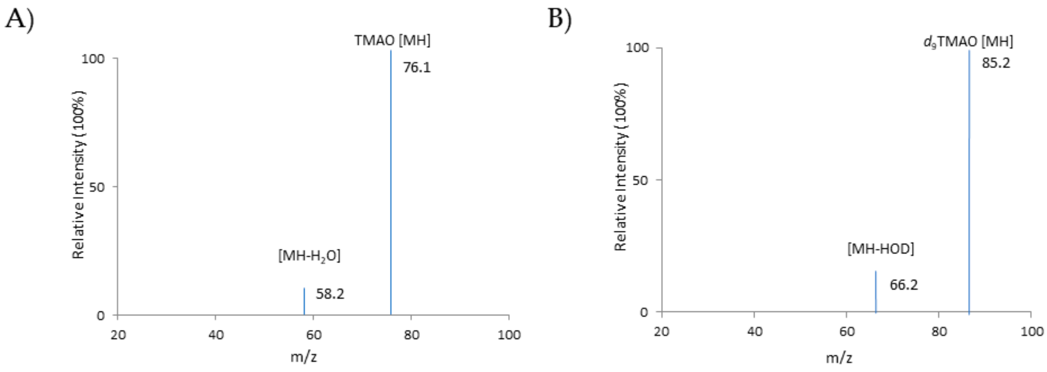

Figure S1. Collision-induced dissociation (CID) spectra of TMAO in tandem mass spectrometry. CID spectrum of plasma (**A**) and the synthetic internal standard of TMAO (**B**) are shown.

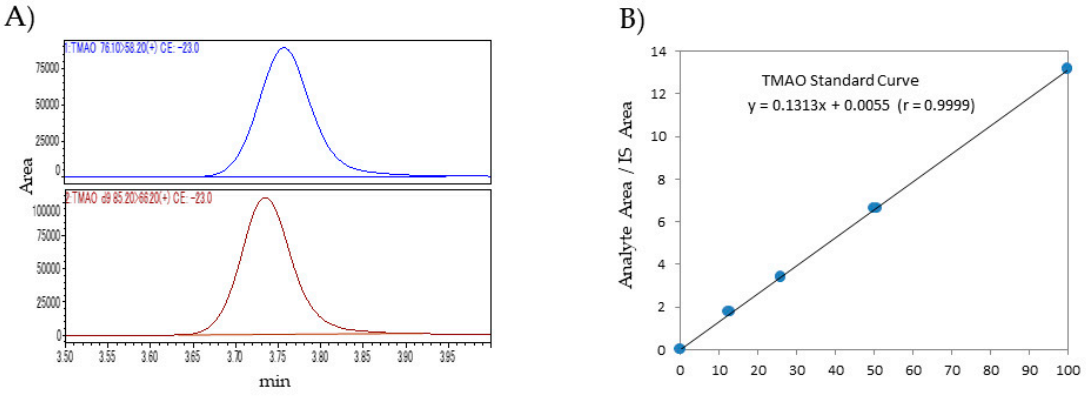

Figure S2. Determination of plasma concentration of TMAO with d9-TMAO as an internal standard using LC-MS/MS. (**A**) Peaks of plasma TMAO (m/z 76) and synthetic d9-TMAO. (**B**) A standard curve of plasma TMAO.
