# Supplementary material for: Neither Trimethylamine-N-Oxide nor Trimethyllysine Is Associated with Atherosclerosis: A Cross-Sectional Study in Older Japanese Adults
Source: Nutrients. 2023 Feb 2;15(3):759. doi: 10.3390/nu15030759 (PMC9921512; doi:10.3390/nu15030759)
Supplement: Supplementary file 1 [file nutrients-15-00759-s001.zip › Table S1.pdf]

Table S1: Correlation analysis of the carnitine-related metabolites in all participants

Model 1	Carnitine	γBB	TMAO	TML
Carnitine	-	0.38**	0.13*	0.23**
γBB	0.38**	-	0.29**	0.33**
TMAO	0.13*	0.29**	-	0.50**
Age	0.02	0.14**	0.08	0.23**
BMI	0.17**	0.02	0.13*	0.10

Pearson's r is indicated in the table. * $p<0.05$, ** $p<0.001$. For this analysis, Pearson's correlation was applied after the log transformation of blood levels of carnitine and the metabolites.
