# Supplementary material for: Neither Trimethylamine-N-Oxide nor Trimethyllysine Is Associated with Atherosclerosis: A Cross-Sectional Study in Older Japanese Adults
Source: Nutrients. 2023 Feb 2;15(3):759. doi: 10.3390/nu15030759 (PMC9921512; doi:10.3390/nu15030759)
Supplement: Supplementary file 1 [file nutrients-15-00759-s001.zip › Table S2.pdf]

**Table S2:** Simple and multiple linear regression analysis for PS in the whole participants.

| Variables | Spearman's ρ | | Model 1 | | | | | | Model 2 | | | | | |
|---|---|---|---|---|---|---|---|---|---|---|---|---|---|---|
| | ρ | p-value | β | SE | Standard β | t | p-value | VIF | β | SE | Standard β | t | p-value | VIF |
| Carnitine | 0.113 | 0.031 | 0.021 | 0.022 | 0.051 | 0.925 | 0.356 | 1.152 | 0.006 | 0.022 | 0.015 | 0.284 | 0.777 | 1.274 |
| γBB | 0.197 | <0.001 | 2.630 | 0.984 | 0.159 | 2.673 | 0.008 | 1.327 | 0.005 | 1.029 | 0.000 | 0.005 | 0.996 | 1.664 |
| TMAO | -0.010 | 0.850 | 0.015 | 0.020 | 0.044 | 0.733 | 0.464 | 1.351 | 0.039 | 0.020 | 0.116 | 1.932 | 0.054 | 1.543 |
| TML | 0.083 | 0.116 | -0.293 | 1.016 | -0.017 | -0.288 | 0.773 | 1.280 | -2.838 | 1.009 | -0.163 | -2.812 | 0.005 | 1.447 |
| Age | 0.316 | <0.001 | | | | | | | 0.122 | 0.025 | 0.262 | 4.886 | <0.001 | 1.234 |
| BMI | 0.021 | 0.619 | | | | | | | -0.037 | 0.057 | -0.035 | -0.647 | 0.518 | 1.223 |
| DL | -0.047 | 0.371 | | | | | | | 0.373 | 0.389 | 0.050 | 0.958 | 0.339 | 1.174 |
| DM | 0.058 | 0.273 | | | | | | | 0.179 | 0.543 | 0.017 | 0.329 | 0.742 | 1.096 |
| HT | 0.235 | <0.001 | | | | | | | 0.597 | 0.395 | 0.080 | 1.511 | 0.132 | 1.212 |
| Drinker | 0.084 | 0.108 | | | | | | | -0.251 | 0.492 | -0.029 | -0.512 | 0.609 | 1.330 |
| Smoker | 0.019 | 0.718 | | | | | | | 0.329 | 0.908 | 0.018 | 0.363 | 0.717 | 1.074 |
| Sex #1 | 0.252 | <0.001 | | | | | | | 2.440 | 0.484 | 0.330 | 5.042 | <0.001 | 1.839 |
| Area #2 | -0.024 | 0.647 | | | | | | | -0.036 | 0.391 | -0.005 | -0.092 | 0.927 | 1.236 |

In Model 1, only the L-carnitine-related metabolites were included in the analysis. In Model 2, all the parameters shown in the left column were included. #1. 0=Women and 1=Men, #2. 0=Kakeya and 1=Oki Island.
