# Supplementary material for: Neither Trimethylamine-N-Oxide nor Trimethyllysine Is Associated with Atherosclerosis: A Cross-Sectional Study in Older Japanese Adults
Source: Nutrients. 2023 Feb 2;15(3):759. doi: 10.3390/nu15030759 (PMC9921512; doi:10.3390/nu15030759)
Supplement: Supplementary file 1 [file nutrients-15-00759-s001.zip › Table S3.pdf]

Table S3 Multiple linear regression analysis for IMT and PS in men.

Variables	IMT						PS					
	β	SE	Standard β	t	*p*-value	VIF	β	SE	Standard β	t	*p*-value	VIF
Carnitine	-0.007	0.009	-0.075	-0.825	0.411	1.221	-0.078	0.043	-0.159	-1.796	0.075	1.221
γBB	0.281	0.432	0.062	0.650	0.517	1.325	2.091	2.080	0.093	1.006	0.316	1.325
TMAO	0.009	0.007	0.125	1.267	0.207	1.424	0.060	0.036	0.159	1.668	0.098	1.424
TML	-0.547	0.313	-0.163	-1.746	0.083	1.293	-3.145	1.508	-0.190	-2.086	0.040	1.293
Age	0.029	0.010	0.268	2.832	0.005	1.319	0.138	0.049	0.257	2.793	0.006	1.319
BMI	-0.024	0.026	-0.084	-0.921	0.359	1.221	-0.187	0.127	-0.130	-1.465	0.145	1.221
DL	0.176	0.191	0.085	0.922	0.358	1.252	1.587	0.920	0.155	1.725	0.087	1.252
DM	0.073	0.221	0.031	0.329	0.743	1.276	-0.119	1.063	-0.010	-0.112	0.911	1.276
HT	0.153	0.179	0.079	0.857	0.393	1.261	1.078	0.860	0.113	1.254	0.212	1.261
Drinker	0.112	0.163	0.061	0.685	0.495	1.151	-0.107	0.786	-0.012	-0.136	0.892	1.151
Smoker	0.020	0.288	0.006	0.068	0.946	1.123	-0.199	1.387	-0.012	-0.144	0.886	1.123
Area #1	0.019	0.171	0.010	0.108	0.914	1.283	-0.238	0.825	-0.026	-0.289	0.773	1.283

#1. 0=Kakeya and 1=Oki island
